# Compositional Phenolic Signatures of Antioxidant-Relevant Compounds in Hop (*Humulus lupulus* L.) Varieties and Local Ecotypes Cultivated in Southern Chile

**DOI:** 10.3390/antiox15040444

**Published:** 2026-04-01

**Authors:** Ignacio Matamala, Manuel Chacón-Fuentes, Daniel Martínez-Cisterna, Pablo Parra-Verdugo, Valeria Asencio-Cancino, Leonardo Bardehle

**Affiliations:** 1 Programa de Doctorado en Ciencias Agroalimentaria y Medioambiente, Facultad de Ciencias Agropecuarias y Medioambiente, Universidad de La Frontera, Av. Francisco Salazar 01145, Casilla 54-D, Temuco 4811230, Chile; ignacio.matamala@ufrontera.cl (I.M.); p.parra03@ufromail.cl (P.P.-V.); v.asencio01@ufromail.cl (V.A.-C.); 2 Departamento de Producción Agropecuaria, Facultad de Ciencias Agropecuarias y Medioambiente, Universidad de La Frontera, Av. Francisco Salazar 01145, Casilla 54-D, Temuco 4811230, Chile; 3 Centro de Genómica Nutricional Agroacuícola (CGNA), Las Heras 350, Temuco 4780000, Chile; 4 Centro de Investigación Biotecnológica Aplicada al Medio Ambiente (CIBAMA), Universidad de La Frontera, Av. Francisco Salazar 01145, Casilla 54-D, Temuco 4811230, Chile; d.martinez11@ufromail.cl; 5 Laboratorio de Entomología Aplicada, Facultad de Ciencias Agropecuarias y Medioambiente, Universidad de La Frontera, Av. Francisco Salazar 01145, Casilla 54-D, Temuco 4811230, Chile

**Keywords:** hop (*Humulus lupulus* L.), phenolic compounds, prenylated flavonoids, xanthohumol, antioxidant capacity, ORAC, phenolic acids, PCA, phenolic allocation indices

## Abstract

Hop (*Humulus lupulus* L.) cones are increasingly recognized as sources of phenolic compounds relevant to antioxidant-oriented applications beyond their traditional brewing role; however, genotype-dependent chemical diversity remains poorly characterized under South American cultivation. This study evaluated phenolic composition and antioxidant-related chemical signatures in 22 hop accessions, including commercial varieties and Chilean local ecotypes, cultivated under homogeneous conditions in southern Chile. Total phenolic content (TPC), total flavonoid content (TFC), and condensed tannins were determined using spectrophotometric assays, while phenolic acids, catechin, and prenylated flavonoids were quantified by HPLC. Antioxidant capacity was evaluated using the ORAC assay, and principal component analysis (PCA) was applied to integrate chemical variables. TPC ranged from 4051 to 8124 mg gallic acid equivalents/100 g dry weight, TFC from 655 to 3011 mg quercetin equivalents/100 g, and condensed tannins from 11.0 to 60.1 mg catechin equivalents/g. ORAC values ranged from 96,405 to 161,815 µmol Trolox equivalents/100 g dry weight, indicating substantial genotype-dependent variation. PCA explained 69.5% of total variance and revealed distinct phenolic composition patterns among genotypes. Pearson correlation analysis showed that antioxidant capacity was strongly associated with condensed tannins and total phenolic content, whereas total flavonoids were not significantly related to ORAC values. Prenylated flavonoids were negatively associated with antioxidant capacity, suggesting a limited contribution to peroxyl radical scavenging activity. These findings highlight the importance of phenolic subclass composition, particularly condensed tannins, in determining antioxidant capacity and support the selection of hop genotypes based on specific phenolic profiles for functional applications.

## 1. Introduction

Phenolic compounds represent a central component of plant secondary metabolism and play an important role in redox-related processes, oxidative regulation, and plant adaptive responses to environmental stress [1,2]. Among these metabolites, flavonoids, phenolic acids, and condensed tannins are particularly relevant due to their structural diversity and their capacity to modulate oxidative processes in biological and food systems [3]. Growing interest in phenolic compounds with redox activity has progressively shifted research from purely quantitative descriptions toward integrative chemical approaches that consider compositional balance, relative allocation among phenolic classes, and multivariate metabolic patterns [4,5,6]. Hop (*Humulus lupulus* L.) is a perennial climbing plant traditionally cultivated for its female inflorescences (cones), which are indispensable in the brewing industry as sources of bitterness, aroma, and microbial stability [7]. Beyond its technological role in beer production, hops have gained increasing recognition as a plant matrix exceptionally rich in phenolic compounds, positioning this species as a promising source of metabolites of chemical relevance in redox-oriented and functional ingredient research contexts beyond brewing [8,9]. Although hop cones are not commonly consumed as a direct food ingredient, their phenolic constituents are partially transferred to beer and have also attracted increasing interest in food science and nutraceutical research as chemically relevant plant-derived metabolites. A distinctive chemical feature of hop cones is the presence of prenylated flavonoids, a relatively rare subclass of flavonoids characterized by the attachment of prenyl side chains to the flavonoid backbone [10,11]. Compounds such as xanthohumol, isoxanthohumol, and related derivatives are largely specific to *H. lupulus* and have been extensively investigated due to their redox-modulating behavior and chemical properties observed in in vitro systems [12]. The prenyl moiety increases lipophilicity and may enhance interactions with hydrophobic environments, which can influence the stability and redox-related behavior of these compounds, conferring physicochemical properties that clearly differentiate them from non-prenylated flavonoids [13]. In addition to prenylated flavonoids, hop cones contain complex mixtures of non-prenylated flavonoids, phenolic acids, and condensed tannins [14]. Phenolic acids such as chlorogenic, caffeic, p-coumaric, and ferulic acids, together with flavonoids including quercetin, kaempferol, and catechin derivatives, contribute to redox-related chemical processes through complementary mechanisms such as free radical scavenging, metal chelation, and redox cycling [15]. Several studies have demonstrated that the relevance of hops in oxidative systems is more closely associated with their overall phenolic composition, particularly prenylated flavonoids, than with bitter acids, which are mainly related to brewing properties. This highlights the importance of comprehensive phenolic profiling rather than single-compound evaluation [16,17,18]. Importantly, redox-related properties of plant materials are not determined solely by absolute concentrations of individual compounds, but also by their relative distribution and compositional balance. In hops, however, such integrative compositional approaches remain underexplored, particularly in comparative studies involving diverse genetic backgrounds cultivated under homogeneous environmental conditions. The accumulation of phenolic compounds in hops is strongly influenced by both genotype and environment [19]. Varietal differences in flavonoid and phenolic acid profiles have been consistently reported, reflecting genotype-dependent regulation of secondary metabolism [20]. Environmental factors such as climate, soil characteristics, and agronomic management further modulate phenolic biosynthesis, leading to pronounced chemical variability across growing regions [21]. Consequently, comparative analyses conducted under identical edaphoclimatic conditions are essential to isolate genetic effects and to identify chemically meaningful patterns among varieties and ecotypes. In recent years, hop cultivation has expanded into non-traditional growing regions, including the Southern Hemisphere. In Chile, the establishment of varietal gardens and the identification of locally adapted hop ecotypes represent a strategic opportunity to diversify hop genetic resources [22,23]. While agronomic adaptation has begun to be documented, detailed chemical characterization of phenolic compounds, particularly prenylated flavonoids and phenolic compositional patterns linked to redox-related properties, in Chilean-grown hops remains limited. However, the relative contribution of different phenolic subclasses to antioxidant capacity in hop remains poorly understood, particularly under controlled environmental conditions. The objective of the present study was to characterize and compare phenolic composition and antioxidant capacity in hop cones from commercial varieties and Chilean local ecotypes cultivated under uniform conditions, with the aim of evaluating genotype-dependent variation in phenolic allocation and associated chemical signatures. The working hypothesis was that hop varieties and local ecotypes grown under identical edaphoclimatic conditions exhibit distinct phenolic allocation patterns and multivariate compositional structures driven primarily by genetic differences, which are reflected in their chemical profiles. By integrating quantitative phenolic data with compositional indices, multivariate analysis, and antioxidant capacity, this study provides a robust chemical framework for understanding the diversity of phenolic metabolites in hops cultivated in an emerging production region and establishes a basis for subsequent functional evaluations.

## 2. Materials and Methods

### 2.1. Plant Material and Experimental Site

Female hop cones (only female plants were included, as male plants do not produce cones and are not used for commercial cone production) were obtained from a varietal hop garden established at the Maquehue Experimental Field, Universidad de La Frontera (Temuco, Chile; 38.8379° S, 72.6938° W; 120 m a.s.l.). The experimental garden comprises a wide genetic diversity of hop material, including internationally recognized commercial cultivars and locally selected Chilean ecotypes. The evaluated hop materials included the following commercial cultivars and local ecotypes: Cascade, Cashmere, Cluster, Crystal, Fuggle, Glacier, Magnum, Northern Brewer, Nugget, Olympic, Perle, Sterling, Styrian Golding, Tahoma, Triumph, Vanguard, Villanette, Yakima Gold, and the Chilean ecotypes Galena, La Unión, Ranco, and Valdivia. All hop accessions were cultivated under homogeneous edaphoclimatic and agronomic conditions (temperate climate with Mediterranean influence, with average annual precipitation of approximately 1200 mm and mean annual temperatures ranging from 10 to 13 °C; soil corresponding to an Andisol of the Temuco series, characterized by volcanic origin, depth up to 1.2 m, loam to clay-loam texture, good permeability and drainage, moderately acidic pH (5.9), low aluminum saturation (1.56%), and adequate available phosphorus levels (37 ppm)) to ensure that observed differences in chemical composition could be attributed primarily to genetic background rather than environmental variability. The varietal garden was designed to allow individual identification and full traceability of each hop accession throughout the growing season. Each cultivar or ecotype was represented by five plants, arranged under a standardized trellis system and managed using uniform cultural practices including standardized planting distances (2.0 m within rows and 2.5 m between rows), mechanical soil preparation prior to establishment, and uniform fertilization using controlled-release fertilizer (Basacote^®^, BASF SE, Ludwigshafen, Germany) supplemented during the growing season. No differential agronomic treatments were applied among varieties or ecotypes during cultivation. Hop cones were harvested manually at technological maturity during the 2025 growing season (corresponding to the reproductive period observed between January and March under local conditions). Harvest timing was determined based on visual assessment of cone development, lupulin gland coloration, and cone texture, following standard criteria for hop technological maturity; no chemical standardization was applied at harvest. For each genotype, cones were collected from the five plants, with approximately 15 cones per plant, and pooled across plants to generate composite samples, from which three independent biological replicates (*n* = 3) were prepared for subsequent chemical analyses. The pooled samples corresponded to approximately 300 g of fresh cone material per biological replicate. Immediately after harvest, cones were subjected to a freeze-drying process using a laboratory freeze dryer (Labconco Corp., Kansas City, MO, USA) to stabilize chemical composition and minimize degradation of phenolic compounds. Samples were frozen at −80 °C prior to drying and subsequently lyophilized under vacuum conditions (≤0.1 mbar) at condenser temperatures of approximately −50 °C for 48 h, following previously described procedures for plant material [24,25]. Dried samples were subsequently stored in airtight polyethylene bags, vacuum-sealed and protected from light, and maintained at 4 °C until further processing and chemical analyses.

### 2.2. Sample Preparation

After harvest, hop cones were subjected to freeze-drying as described in Section 2.1. Lyophilized cones were manually inspected and cleaned to remove leaves, stems, and other extraneous plant material that could interfere with chemical determinations. Only intact female cones were selected to ensure sample homogeneity and comparability among hop varieties and ecotypes. Cleaned, lyophilized cones were ground to a fine and homogeneous powder using a laboratory-scale mill (Retsch Grindomix GM 200, Haan, Germany). Grinding was conducted under controlled conditions (short grinding cycles to avoid heat buildup) to minimize heat generation and prevent potential degradation of phenolic compounds. The resulting powders were passed through a standardized stainless steel sieve (500 µm mesh size) to ensure uniform particle size distribution, which is critical for extraction efficiency and analytical reproducibility. Ground samples were stored in airtight polyethylene containers, protected from light, and maintained at 4 °C under refrigerated conditions, minimizing exposure to oxygen and moisture until chemical analyses were performed. Storage duration prior to analysis did not exceed 7 days to minimize the risk of oxidative or hydrolytic degradation of phenolic constituents. For each hop variety or ecotype, independent sample replicates were prepared to account for within-accession variability, with each replicate originating from an independently prepared batch of lyophilized and ground material.

### 2.3. Extraction of Phenolic Compounds

Phenolic compounds were extracted from lyophilized hop cone powders using a hydroalcoholic extraction protocol based on previously reported methodologies for phenolic compound extraction in plant matrices [26], applied to both spectrophotometric and chromatographic analyses. Briefly, 1.0 g of lyophilized and finely ground hop sample was extracted with 20 mL of 80% (*v*/*v*) methanol acidified with 0.1% (*v*/*v*) HCl. The extraction mixture was subjected to ultrasonic-assisted extraction for 30 min at a frequency of 37 kHz, while maintaining the temperature between 20 and 30 °C and protecting samples from light to minimize oxidation and degradation of phenolic compounds. Following sonication, extracts were centrifuged at 4000 rpm for 10 min, and the resulting supernatants were collected. Extracts were subsequently filtered through 0.22 μm PVDF membrane filters (Merck Millipore, Burlington, MA, USA) to remove particulate matter prior to analysis. Filtered extracts were used directly or appropriately diluted according to the analytical requirements of each assay [27]. Extracts obtained using this protocol were employed for the determination of total phenolic content, total flavonoid content, condensed tannins, and HPLC-based quantification of prenylated flavonoids and phenolic acids, thereby ensuring consistency and comparability between spectrophotometric and chromatographic analyses. For spectrophotometric assays, extracts were diluted with the extraction solvent to obtain absorbance values within the linear range of the corresponding calibration curves. For chromatographic analyses, filtered extracts were transferred to HPLC vials and injected without further modification. All extractions were performed using independent biological replicates for each hop variety or ecotype, with each replicate originating from an independently prepared lyophilized sample, thereby accounting for within-accession variability and ensuring analytical robustness.

### 2.4. Determination of Total Phenolic Content

Total phenolic content (TPC) was determined using the Folin–Ciocalteu colorimetric method, following a protocol commonly applied to hop samples. Briefly, 20 μL of appropriately diluted hop extract was mixed with 1580 μL of distilled water and 100 μL of Folin–Ciocalteu reagent. After thorough mixing, 300 μL of sodium carbonate solution (20% *w*/*v*) was added to the reaction mixture, resulting in a final reaction volume of 2000 μL. The reaction mixtures were vortexed and incubated for 2 h at room temperature in the dark to allow complete color development. Following incubation, 300 μL of each reaction mixture was transferred to a 96-well microplate, and absorbance was measured at 765 nm using a BioTek Synergy™ HTX multimode microplate reader (BioTek Instruments, Inc., Winooski, VT, USA). Absorbance values were maintained within the linear range of the assay (0.1–0.8 absorbance units) to ensure compliance with the Beer–Lambert law. Gallic acid was used as the calibration standard, and standard solutions were prepared at concentrations ranging from 0 to 800 mg L^−1^. A calibration curve was constructed by plotting absorbance against gallic acid concentration, and total phenolic content was calculated by interpolation. Results were expressed as milligrams of gallic acid equivalents (mg GAE) per 100 g of dry sample. All measurements were performed using extracts obtained from independent biological replicates for each hop variety or ecotype, and each extract was analyzed in analytical triplicate to ensure reproducibility and analytical reliability [28].

### 2.5. Determination of Total Flavonoid Content

Total flavonoid content (TFC) was determined using the aluminum chloride colorimetric method, following a protocol commonly applied to hop extracts. Briefly, 250 μL of appropriately diluted extract was mixed with 1250 μL of distilled water. Subsequently, 75 μL of sodium nitrite solution (5% *w*/*v*) was added, and the mixture was allowed to react for 5 min at room temperature. Then, 150 μL of aluminum chloride solution (10% *w*/*v*) was added, and the reaction was incubated for an additional 6 min. After this period, 500 μL of sodium hydroxide solution (1 M) was added to stop the reaction and promote color development. The final reaction volume was adjusted to 2500 μL with distilled water, and the mixture was thoroughly vortexed. After 15 min of incubation at room temperature, 300 μL of each reaction mixture was transferred to a 96-well microplate, and absorbance was measured at 510 nm using a BioTek Synergy™ HTX multimode microplate reader. Quercetin was used as the calibration standard, and standard solutions were prepared over a concentration range of 0 to 1000 mg L^−1^. A calibration curve was constructed by plotting absorbance against quercetin concentration, and total flavonoid content was calculated by interpolation [29]. Results were expressed as milligrams of quercetin equivalents (mg QE) per 100 g of dry sample. All determinations were performed using extracts obtained from independent biological replicates for each hop variety or ecotype, and each extract was analyzed in analytical triplicate to ensure accuracy and reproducibility of the measurements.

### 2.6. Determination of Condensed Tannins

Condensed tannin content was determined using the vanillin–HCl colorimetric method, as commonly applied to hop extracts. Briefly, 50 μL of appropriately diluted extract was mixed with 150 μL of vanillin reagent (1% *w*/*v* vanillin in methanol). Subsequently, 750 μL of hydrochloric acid solution (8% *v*/*v* in methanol) was added to initiate the reaction. The reaction mixtures were vortexed and incubated for 20 min at room temperature in the dark to allow complete color development. Following incubation, 300 μL of each reaction mixture was transferred to a 96-well microplate, and absorbance was measured at 500 nm using a BioTek Synergy™ HTX multimode microplate reader. Blanks were prepared by replacing the vanillin reagent with methanol to correct for background absorbance. Catechin was used as the reference standard, and standard solutions were prepared over a concentration range of 0 to 1000 mg L^−1^. A calibration curve was constructed by plotting absorbance against catechin concentration, and condensed tannin content was calculated by interpolation. Results were expressed as milligrams of catechin equivalents (mg CE) per gram of dry sample [30]. All measurements were performed using extracts obtained from independent biological replicates for each hop variety or ecotype, and each extract was analyzed in analytical triplicate to ensure analytical precision and reproducibility.

### 2.7. HPLC Analysis

#### 2.7.1. HPLC Analysis of Phenolic Acids and Individual Flavonoids

Phenolic acids and individual flavonoids were analyzed by high-performance liquid chromatography coupled to diode-array detection (HPLC–DAD; Shimadzu, Kyoto, Japan). Chromatographic separation was performed using a LC-2050C 3D liquid chromatograph (Shimadzu, Kyoto, Japan) equipped with a PDA detector (1024 diodes). Separation was achieved on a reversed-phase RP-C18 Isis column (250 × 4.6 mm, 5 μm) coupled to a NUCLEODUR C18 Isis guard column (EC 4/3, 5 μm). The mobile phase consisted of solvent A (3% *v*/*v* acetic acid in water) and solvent B, composed of 3% (*v*/*v*) acetic acid, acetonitrile, and water (3:25:72, *v*/*v*/*v*). Elution was carried out at a constant flow rate of 1.0 mL min^−1^ using a gradient program starting at 100% A and 0% B, maintained for 5 min, followed by a gradual increase in solvent B from 10% to 20% between 5.01 and 10.01 min. Subsequently, solvent B was increased stepwise up to 100% at 75 min. The system was then returned to the initial conditions, and the column was allowed to re-equilibrate until the end of the run, resulting in a total analysis time of 90 min. Prior to chromatographic analysis, hop extracts were prepared as described in Section 2.3 and filtered through 0.22 μm hydrophilic PVDF membrane filters. Identification of phenolic acids and individual flavonoids was performed by comparing retention times and UV–visible spectra with those of authentic standards. Quantification was carried out using external calibration curves constructed with available analytical standards, and results were expressed as mg g^−1^ dry weight. Calibration curves were evaluated for linearity within the working range [31].

#### 2.7.2. HPLC Analysis of Prenylated Flavonoids

Prenylated flavonoids were analyzed by high-performance liquid chromatography coupled to diode-array detection (HPLC–DAD). Chromatographic separation was performed using a MultoKrom RP-18e C18 column (250 × 4.6 mm, 5 μm; CS-Chromatographie Service GmbH, Langerwehe, Germany) maintained at 30 °C. The mobile phase consisted of solvent A (ultrapure water acidified with 0.25% *v*/*v* formic acid) and solvent B (acetonitrile acidified with 0.25% *v*/*v* formic acid). Elution was carried out at a constant flow rate of 1.0 mL min^−1^ with an injection volume of 20 μL. The gradient program started at 80% A and 20% B and was maintained for 3 min, followed by a linear increase to 75% B at 35 min. This condition was held until 37 min, after which the system was returned to the initial conditions (80% A and 20% B) at 40 min, with column re-equilibration until 45 min. Detection was performed using diode-array detection with selective monitoring at 370 nm for xanthohumol (XN) and desmethylxanthohumol (DMX), and at 290–292 nm for isoxanthohumol (IXN), 8-prenylnaringenin (8-PN), and 6-prenylnaringenin (6-PN). Identification was based on retention times and UV–visible spectra compared with those of authentic standards. Quantification was performed using external calibration with naringenin as the reference standard, combined with compound-specific response correction factors for prenylated flavonoids lacking available analytical standards. Correction factors of 0.583 for XN and 1.296 for IXN, 8-PN, and 6-PN were applied according to previously reported methodology. Calibration curves were evaluated for linearity within the working range. Concentrations were calculated by interpolation from calibration curves and corrected using the corresponding response factors. Final results were expressed as mg g^−1^ dry weight [31,32].

### 2.8. Antioxidant-Related Chemical Indices

Chemical indices were calculated to integrate spectrophotometric and chromatographic data and to describe relative chemical allocation patterns among hop varieties and local ecotypes. These indices were derived from quantitative results obtained for total phenolics, total flavonoids, phenolic acids, and prenylated flavonoids and were used as comparative compositional descriptors rather than absolute stoichiometric measures. These indices represent novel compositional descriptors proposed to describe relative phenolic allocation patterns. The prenylation index was calculated as the ratio between the total concentration of prenylated flavonoids quantified by HPLC and the total flavonoid content determined by the aluminum chloride colorimetric assay, according to the following equation:

**(1)**  **Prenylation index:**

Prenylation index = (Σ Prenylated flavonoids)/(Total flavonoids)

where the sum of prenylated flavonoids corresponds to the combined concentrations of xanthohumol, desmethylxanthohumol, isoxanthohumol, 8-prenylnaringenin, and 6-prenylnaringenin, expressed on a dry weight basis.

The phenolic acid index was calculated as the ratio between the total concentration of phenolic acids identified by HPLC and the total phenolic content determined by the Folin–Ciocalteu assay, as shown in the following equation:

**(2)**  **Phenolic acid index**

Phenolic acid index = (Σ Phenolic acids)/(Total phenolics)

where the sum of phenolic acids includes all individual phenolic acids quantified by HPLC–DAD.

Because these indices combine data derived from analytical methods with different response characteristics, they should be interpreted as semi-quantitative compositional indicators reflecting relative allocation among phenolic subclasses rather than as absolute chemical proportions. Their primary purpose is to enable comparative assessment of phenolic distribution patterns among hop genotypes cultivated under identical conditions.

Index values range between 0 and 1, where values approaching 1 indicate a greater relative allocation of the numerator phenolic subclass within the corresponding compositional pool, whereas values closer to 0 reflect lower proportional representation.

In addition, relative contribution values used for stacked bar representations were calculated by normalizing the sum of prenylated flavonoids and phenolic acids to their combined total, as follows:

**(3)**  **Relative contribution of prenylated flavonoids**

Relative prenylated flavonoids = (Σ Prenylated flavonoids)/(Σ Prenylated flavonoids + Σ Phenolic acids),

**(4)**  **Relative contribution of phenolic acids**

Relative phenolic acids = (Σ Phenolic acids)/(Σ Prenylated flavonoids + Σ Phenolic acids)

All indices were calculated for each biological replicate and are expressed as dimensionless ratios. Replicate-level values were used for comparative visualization, one-way ANOVA, and multivariate analyses, including principal component analysis.

### 2.9. Determination of Antioxidant Capacity by ORAC Assay

Antioxidant capacity was evaluated using the oxygen radical absorbance capacity (ORAC) assay. Extracts obtained as described in Section 2.3 were diluted in phosphate buffer (75 mM, pH 7.4) to obtain appropriate working concentrations within the linear range of the assay. For the assay, 150 µL of fluorescein solution (80 nM) was added to each well containing the diluted extract and incubated at 45 °C for 30 min. The reaction was initiated by adding 25 µL of AAPH solution (150 mM), which generates peroxyl radicals. Fluorescence decay was monitored over 90 min at 1-min intervals using a microplate reader (BioTek Synergy™ HTX; BioTek Instruments, Inc., Winooski, VT, USA) at excitation 485/20 nm and emission 520 nm. Trolox was used as an external standard. A stock solution (4834.39 µmol L^−1^) was prepared, and calibration points were generated by adding 5, 10, 20, and 40 µL of the standard solution. The resulting calibration curve showed high linearity (R^2^ = 0.995). Antioxidant capacity was calculated based on the net area under the fluorescence decay curve (AUC), after subtraction of the blank, and expressed as µmol Trolox equivalents per 100 g dry weight (µmol TE 100 g^−1^ DW) [33]. ORAC values were obtained from three independent biological replicates per genotype, each measured in analytical triplicate. Technical replicates were averaged prior to statistical analysis, and the resulting biological replicate means were used as the experimental units for genotype comparisons.

### 2.10. Statistical and Multivariate Analysis

All quantitative results obtained from spectrophotometric assays (total phenolics, total flavonoids, and condensed tannins) and chromatographic determinations (individual phenolic acids, flavonoids, and prenylated flavonoids) were expressed as mean ± standard error (SE) based on independent biological replicates (*n* = 3). Prior to statistical analysis, data were tested for normality using the Shapiro–Wilk test and for homogeneity of variances using Levene’s test. When both assumptions were met, differences among hop varieties and local ecotypes were evaluated using one-way analysis of variance (ANOVA), considering hop material (variety/ecotype) as a fixed factor. When significant effects were detected (*p* < 0.05), means were separated using Tukey’s honestly significant difference (HSD) test. Results are reported using letter groupings, where means sharing at least one letter are not significantly different at *p* < 0.05. To explore multivariate relationships and compositional differentiation among hop genotypes, principal component analysis (PCA) was performed using autoscaled (z-score standardized) variables. PCA was conducted on standardized mean values of total phenolic content, total flavonoid content, condensed tannins, chemical indices, and major prenylated flavonoids. The analysis was used to identify patterns of variation among genotypes and to determine the variables contributing most strongly to sample separation, which was visualized using score and loading biplots. Pearson’s correlation analysis was performed to assess the relationships between antioxidant capacity (ORAC) and phenolic-related variables, including total phenolic content, total flavonoid content, condensed tannins, total prenylated flavonoids, and individual prenylated flavonoids (xanthohumol, desmethylxanthohumol, isoxanthohumol, 8-prenylnaringenin, and 6-prenylnaringenin). Correlations were calculated using genotype mean values. Total prenylated flavonoids were estimated as the sum of individual compounds. Correlation coefficients (r) and corresponding *p*-values were obtained, and significance was set at *p* < 0.05. All statistical analyses were conducted at a significance level of α = 0.05.

## 3. Results

### 3.1. Total Phenolics, Total Flavonoids, and Condensed Tannins

#### 3.1.1. Total Phenolic Content

Total phenolic content (TPC) differed significantly among hop varieties and local ecotypes cultivated under identical conditions (F = 820, *p* < 0.0001; Table 1). TPC values ranged from 4050.98 ± 32.13 to 8123.83 ± 65.48 mg GAE/100 g DW, indicating substantial genotype-dependent variability in phenolic accumulation. The highest phenolic content was observed in the Valdivia ecotype (8123.83 ± 65.48 mg GAE/100 g DW), followed by high-performing commercial cultivars such as Styrian Golding (7539.76 ± 25.54 mg GAE/100 g DW) and Cluster (7206.01 ± 0.00 mg GAE/100 g DW). In contrast, the lowest TPC values were recorded in Nugget (4050.98 ± 32.13 mg GAE/100 g DW) and Triumph (4252.34 ± 29.58 mg GAE/100 g DW). Overall, both commercial varieties and local ecotypes exhibited a wide range of phenolic content, with partially overlapping distributions. However, the highest TPC values were predominantly associated with specific genotypes, including locally adapted ecotypes, highlighting the influence of genetic background on phenolic accumulation (Table 2).

#### 3.1.2. Total Flavonoid Content

Total flavonoid content (TFC) also differed significantly among hop genotypes (F = 310, *p* < 0.0001; Table 1), ranging from 655.27 ± 15.55 to 3011.28 ± 21.47 mg QE/100 g DW. In contrast to total phenolics, the distribution of flavonoids did not follow the same ranking pattern across hop materials, indicating differences in the relative contribution of flavonoid subclasses. The highest TFC values were observed in commercial cultivars such as Glacier (3011.28 ± 21.47 mg QE/100 g DW), Tahoma (2947.80 ± 12.08 mg QE/100 g DW), and Sterling (2810.74 ± 34.16 mg QE/100 g DW), whereas the lowest values were recorded in Nugget (655.27 ± 15.55 mg QE/100 g DW) and Perle (663.59 ± 11.57 mg QE/100 g DW). Local ecotypes generally exhibited intermediate TFC values, with La Unión (2171.91 ± 47.24 mg QE/100 g DW) showing the highest flavonoid content among them. Notably, the distribution of TFC did not strictly parallel that of total phenolics, as observed in genotypes such as the Valdivia ecotype, which exhibited high TPC but only moderate TFC values. This pattern is consistent with the known selectivity of the aluminum chloride assay, which preferentially detects flavonols and flavones rather than flavan-3-ols, suggesting that differences in flavonoid composition, rather than total abundance alone, contribute to the observed variability. Overall, commercial cultivars tended to reach higher maximum flavonoid concentrations than local ecotypes, although substantial overlap in TFC values was observed between both groups (Table 2).

#### 3.1.3. Condensed Tannin Content

Condensed tannin content also differed significantly among hop genotypes (F = 62.96, *p* < 0.0001; Table 1), ranging from 10.98 ± 0.47 to 60.05 ± 0.81 mg CE/g DW, indicating substantial variability in tannin accumulation. The distribution of condensed tannins did not parallel the patterns observed for total phenolics or total flavonoids, further supporting differences in phenolic subclass allocation among genotypes. The highest tannin content was recorded in the Valdivia ecotype (60.05 ± 0.81 mg CE/g DW), followed by high values in commercial cultivars such as Cluster (56.29 ± 0.53 mg CE/g DW) and Tahoma (43.83 ± 2.55 mg CE/g DW). In contrast, the lowest levels were observed in Yakima Gold (10.98 ± 0.47 mg CE/g DW) and Nugget (12.95 ± 0.48 mg CE/g DW). Local ecotypes exhibited contrasting tannin profiles, with some genotypes reaching levels comparable to or exceeding those observed in commercial cultivars. Overall, the combined assessment of total phenolics, total flavonoids, and condensed tannins reveals substantial compositional variability among hop genotypes cultivated under uniform conditions, highlighting the strong influence of genetic background on phenolic accumulation (Table 2). 

### 3.2. Phenolic Acids and Individual Flavonoids Determined by HPLC

HPLC analysis revealed significant quantitative differences in individual phenolic acids among hop genotypes (*p* < 0.0001). Overall, phenolic acids showed consistent genotype-dependent patterns, with concentrations generally ranging between 0.05 and 0.63 mg g^−1^ DW depending on the compound. Gallic, protocatechuic, and caffeic acids were the predominant phenolic acids across all samples, exhibiting similar distribution patterns among genotypes. The highest concentrations for these compounds were consistently observed in the Valdivia ecotype (0.52 ± 0.02, 0.63 ± 0.02, and 0.47 ± 0.02 mg g^−1^ DW for gallic, protocatechuic, and caffeic acids, respectively), followed by high-performing commercial cultivars such as Cluster and Styrian Golding. In contrast, lower concentrations were generally found in cultivars such as Perle and Nugget. Minor phenolic acids, including vanillic, syringic, and p-coumaric acids, were detected at lower concentrations but followed comparable genotype-dependent trends, with maximum values again associated with the Valdivia ecotype. Ellagic acid showed a similar pattern, with values ranging from 0.07 ± 0.00 to 0.30 ± 0.01 mg g^−1^ DW, reaching its highest levels in Valdivia. Overall, the HPLC results indicate a coherent compositional pattern across phenolic acids, with certain genotypes, particularly the Valdivia ecotype and selected commercial cultivars such as Cluster and Styrian Golding, consistently exhibiting higher accumulation of multiple phenolic acids. These findings suggest coordinated regulation of phenolic acid biosynthesis and contribute to the compositional differentiation observed among hop genotypes (Table 3).

HPLC quantification of individual flavonoids revealed clear genotype-dependent differences among hop genotypes (*p* < 0.0001). Catechin was the predominant individual flavonoid detected across all samples, with concentrations ranging from 0.31 ± 0.01 to 0.83 ± 0.03 mg g^−1^ DW, indicating substantial variability in flavonoid allocation. The highest catechin levels were consistently observed in the Valdivia ecotype (0.83 ± 0.03 mg g^−1^ DW), followed by both local ecotypes and commercial cultivars such as La Unión (0.76 ± 0.02 mg g^−1^ DW), Ranco (0.72 ± 0.02 mg g^−1^ DW), and Cluster (0.81 ± 0.03 mg g^−1^ DW). In contrast, the lowest concentrations were recorded in Perle (0.31 ± 0.01 mg g^−1^ DW) and Nugget (0.33 ± 0.01 mg g^−1^ DW). Notably, the distribution of catechin did not strictly parallel total flavonoid content, as some genotypes with moderate TFC values exhibited relatively high catechin concentrations. This pattern reflects differences in flavonoid subclass composition, particularly the contribution of flavan-3-ols, which are less responsive to the aluminum chloride assay used for TFC determination. Overall, both commercial cultivars and local ecotypes exhibited overlapping catechin ranges, although several ecotypes tended to show elevated concentrations, reinforcing the presence of genotype-dependent differences in flavonoid composition (Table 3).

### 3.3. Prenylated Flavonoids Determined by HPLC

HPLC analysis revealed pronounced quantitative differences in the accumulation of prenylated flavonoids among hop genotypes (*p* < 0.0001). Xanthohumol, desmethylxanthohumol, isoxanthohumol, 8-prenylnaringenin, and 6-prenylnaringenin were detected in all hop materials; however, their concentrations varied markedly across genotypes, indicating strong genotype-dependent differences in prenylflavonoid accumulation.

#### 3.3.1. Xanthohumol and Desmethylxanthohumol

Xanthohumol was the predominant prenylated flavonoid detected in all hop genotypes (Table 4), with concentrations ranging from 0.49 ± 0.01 to 6.01 ± 0.07 mg g^−1^ DW, indicating pronounced genotype-dependent variability. The highest levels were observed in Vanguard (6.01 ± 0.07 mg g^−1^ DW), followed by Magnum (4.83 ± 0.06 mg g^−1^ DW) and Crystal (3.44 ± 0.05 mg g^−1^ DW), whereas markedly lower concentrations were detected in Yakima Gold (0.51 ± 0.01 mg g^−1^ DW) and Cluster (0.63 ± 0.01 mg g^−1^ DW). Local ecotypes exhibited intermediate xanthohumol levels, with Valdivia (1.72 ± 0.02 mg g^−1^ DW), La Unión (1.54 ± 0.02 mg g^−1^ DW), and Ranco (1.39 ± 0.02 mg g^−1^ DW) falling within the mid-range of the evaluated materials. Desmethylxanthohumol followed a similar genotype-dependent pattern, with concentrations ranging from 0.15 ± 0.00 to 2.04 ± 0.02 mg g^−1^ DW. The highest values were again recorded in Vanguard (2.04 ± 0.02 mg g^−1^ DW), followed by Magnum (1.63 ± 0.02 mg g^−1^ DW) and Crystal (1.21 ± 0.02 mg g^−1^ DW), while the lowest concentrations were observed in Cluster and the Galena ecotype (0.15 ± 0.00 mg g^−1^ DW).

#### 3.3.2. Isoxanthohumol and Prenylnaringenins

Isoxanthohumol concentrations varied markedly among hop genotypes, ranging from non-detectable levels to 0.61 ± 0.01 mg g^−1^ DW (Table 4). The highest values were recorded in the Ranco ecotype (0.61 ± 0.01 mg g^−1^ DW), followed by Nugget and the La Unión ecotype (both 0.55 ± 0.01 mg g^−1^ DW), whereas several genotypes exhibited negligible or non-detectable levels. Among prenylnaringenins, 8-prenylnaringenin was detected at low to moderate concentrations, reaching a maximum of 0.63 ± 0.01 mg g^−1^ DW in Nugget and the Ranco ecotype, followed by Glacier (0.61 ± 0.01 mg g^−1^ DW). In contrast, 6-prenylnaringenin was consistently detected at very low levels, with maximum values of 0.07 ± 0.00 mg g^−1^ DW observed in Nugget, Tahoma, and Vanguard, while several genotypes showed no detectable concentrations. Overall, the prenylated flavonoid profile revealed clear genotype-dependent differentiation. Commercial cultivars tended to exhibit higher concentrations of xanthohumol and desmethylxanthohumol, whereas local ecotypes displayed distinctive accumulation patterns for isoxanthohumol and prenylnaringenins, highlighting differences in prenylation and transformation pathways under uniform cultivation conditions. Naringenin, included as the non-prenylated flavanone precursor, was detected at low concentrations across all genotypes and showed relatively limited variation compared to its prenylated derivatives, supporting its role as a structural precursor rather than a major accumulating compound.

### 3.4. Derived Phenolic and Antioxidant Indices

The relative contribution of prenylated flavonoids and phenolic acids across hop genotypes revealed substantial genotype-dependent variation in the proportional allocation between both phenolic subclasses. Relative prenylated flavonoid contributions ranged from 0.07 to 0.90. The highest relative contribution of prenylated flavonoids was observed in Vanguard (0.90), followed by Glacier (0.77) and several cultivars with values above 0.70, indicating a strong predominance of prenylated compounds. In contrast, Cluster (0.07) exhibited the lowest relative contribution of prenylated flavonoids, reflecting a marked dominance of phenolic acids. Similar patterns of high phenolic acid contribution were observed in genotypes such as La Unión and Styrian Golding. Several hop materials displayed intermediate allocation patterns, including Crystal (0.53) and Valdivia (0.54), while Cascade (0.50) showed a balanced distribution between both phenolic subclasses. Overall, these results reveal pronounced genotype-dependent differences in phenolic allocation, highlighting a continuum from prenylated flavonoid-dominated to phenolic acid-dominated profiles. This variation reflects differential metabolic allocation among phenolic subclasses and contributes to the compositional diversity observed among hop genotypes cultivated under uniform conditions.

**Figure 1:**
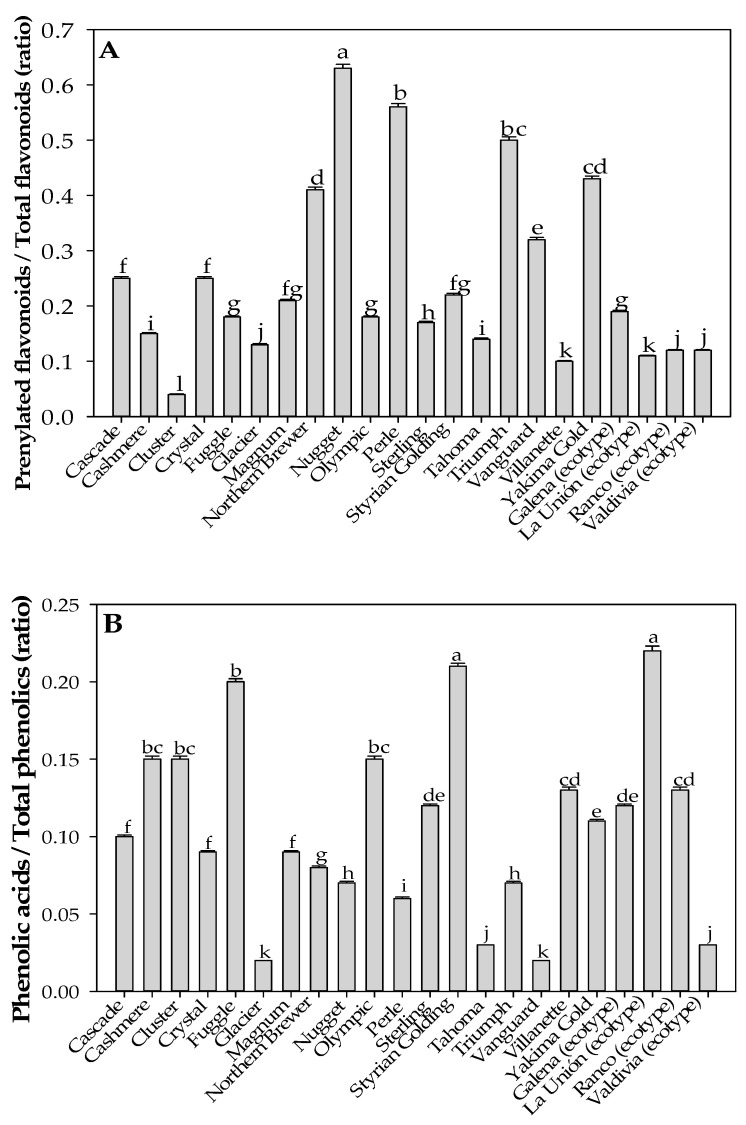
Chemical indices derived from phenolic composition in hop varieties and local ecotypes. (**A**) Ratio of prenylated flavonoids to total flavonoids, calculated from HPLC-determined prenylated flavonoids and spectrophotometrically determined total flavonoid content. (**B**) Ratio of phenolic acids to total phenolics, calculated from the sum of phenolic acids identified by HPLC and spectrophotometrically determined total phenolic content. Values are expressed as mean ± standard error (SE) (*n* = 3). Different letters above the bars indicate significant differences among hop genotypes according to one-way ANOVA followed by Tukey’s multiple comparison test (*p* < 0.05).

Figure 1 illustrates the distribution of antioxidant-relevant chemical indices across hop genotypes. The prenylation index (total prenylated flavonoids/total flavonoids; Figure 1A) exhibited strong genotype-dependent variation, spanning more than one order of magnitude across hop materials. High prenylation index values were observed in genotypes such as Nugget, Perle, and Triumph, as well as Yakima Gold and Northern Brewer, indicating a greater relative allocation of prenylated flavonoids. In contrast, lower values were recorded in Cluster, Villanette, and several local ecotypes, including La Unión, Ranco, and Valdivia, reflecting a reduced contribution of prenylated compounds to the total flavonoid pool. Several commercial cultivars displayed intermediate values, highlighting a continuum of allocation patterns. The phenolic acid index (total phenolic acids/total phenolic content; Figure 1B) also varied substantially among genotypes. The highest ratios were observed in La Unión and Styrian Golding, followed by Fuggle, whereas lower values were detected in Glacier and Vanguard, indicating reduced relative contributions of phenolic acids. Most remaining genotypes exhibited intermediate values. Overall, these indices demonstrate that hop genotypes differ not only in absolute phenolic abundance but also in the proportional allocation among phenolic subclasses. This integrative approach reveals distinct compositional strategies and provides a robust framework for interpreting the multivariate patterns observed in subsequent analyses.

### 3.5. Principal Component Analysis (PCA)

**Figure 2:**
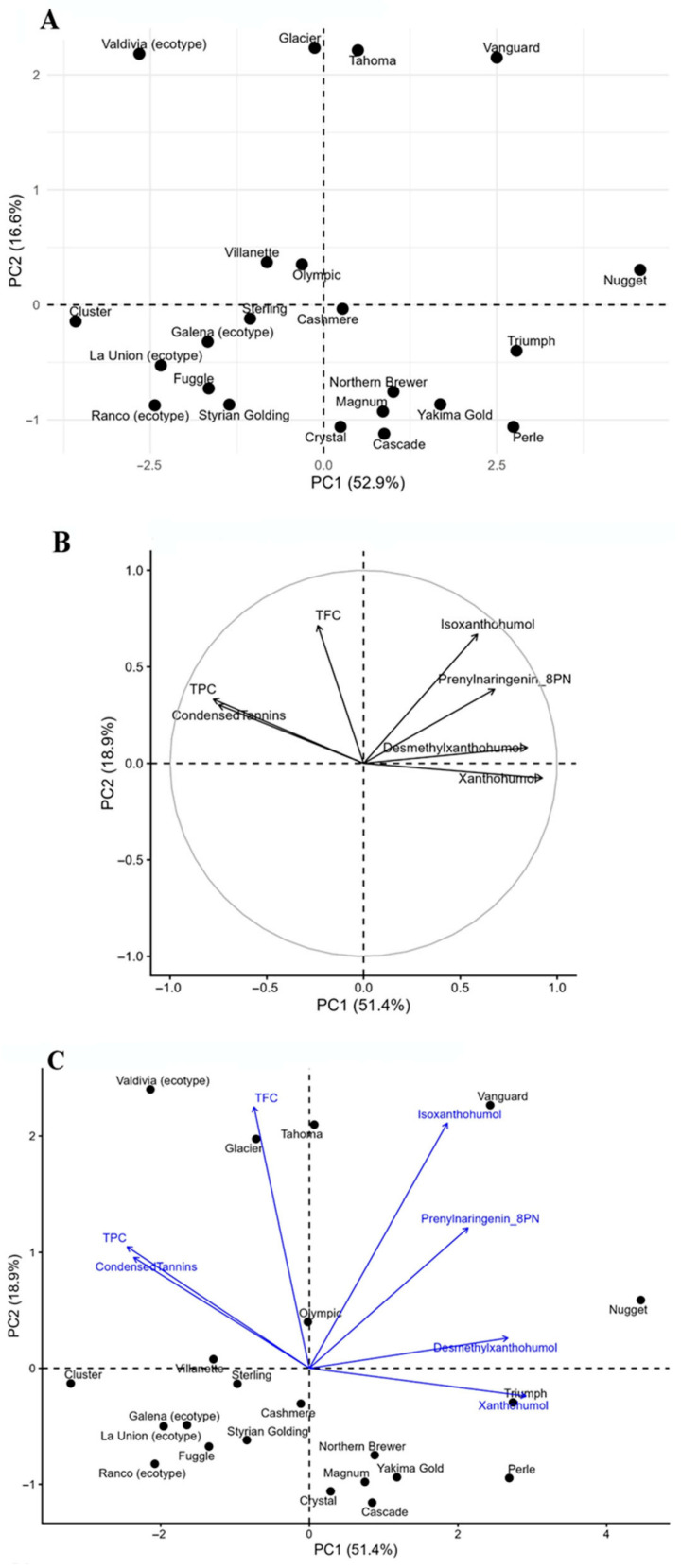
Multivariate analysis of phenolic composition in hop varieties and local ecotypes. (**A**) Principal component analysis score plot showing the distribution of hop genotypes based on phenolic composition. (**B**) PCA loading plot indicating the contribution of individual variables to the principal components. The circle represents the correlation circle; variables closer to the circumference are better represented by PC1 and PC2. (**C**) PCA biplot integrating sample distribution and variable contributions. The analysis included TPC, TFC, condensed tannins, prenylation index, phenolic acid index, and the major prenylated flavonoids quantified by HPLC–DAD (xanthohumol, desmethylxanthohumol, isoxanthohumol, and 8-prenylnaringenin).

Principal component analysis (PCA) was conducted using TPC, TFC, condensed tannins, prenylation index, phenolic acid index, and the major quantified prenylated flavonoids (xanthohumol, desmethylxanthohumol, isoxanthohumol, and 8-prenylnaringenin) to explore multivariate relationships among hop genotypes (Figure 2A–C). The first two principal components explained a large proportion of the total variance, with PC1 accounting for 52.9% and PC2 for 16.6%. The PCA score plot (Figure 2A) revealed clear genotype-dependent distribution patterns. Genotypes located on the positive side of PC1, including Nugget, Perle, Triumph, and Vanguard, were associated with higher levels of prenylated flavonoids. In contrast, genotypes positioned on the negative side of PC1, such as Cluster, La Unión, Ranco, and Fuggle, were characterized by lower prenylated flavonoid contributions and a greater influence of phenolic acids.

The loading plot (Figure 2B) showed that xanthohumol, desmethylxanthohumol, isoxanthohumol, and 8-prenylnaringenin contributed strongly and positively to PC1, confirming that this axis represents a gradient of prenylated flavonoid accumulation. In contrast, TPC and condensed tannins were associated with the negative side of PC1, indicating an opposing contribution of bulk phenolic variables and condensed tannins. PC2 was mainly associated with variation in TFC, contributing to the vertical separation of genotypes. For example, Valdivia, Glacier, and Tahoma were positioned at higher PC2 values, reflecting higher relative flavonoid contributions, whereas genotypes such as Crystal and Cascade showed lower PC2 scores. The PCA biplot (Figure 2C) further confirmed these relationships, illustrating the association between genotype distribution and the contribution of individual phenolic variables. Overall, the PCA revealed a clear separation between genotypes dominated by prenylated flavonoids and those characterized by higher relative contributions of phenolic acids and bulk phenolic parameters.

**Figure 3:**
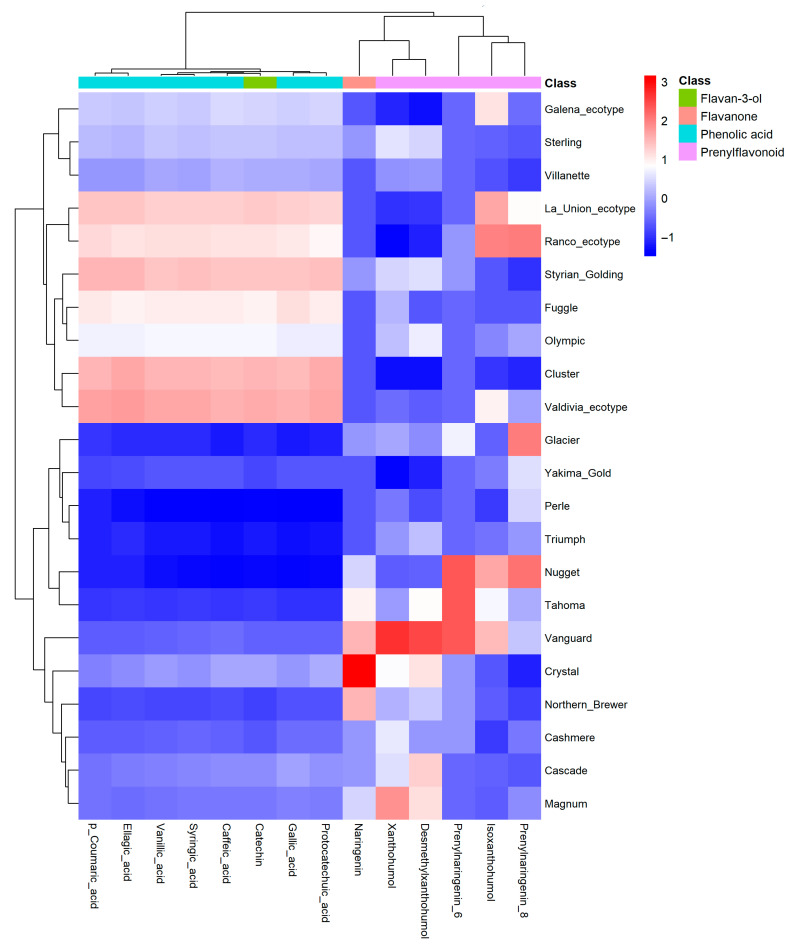
Heatmap and hierarchical clustering of phenolic compounds in hop varieties and local ecotypes based on HPLC–DAD data. Color scale represents normalized concentration values (z-scores), highlighting relative accumulation patterns of phenolic acids, flavonoids, and prenylated flavonoids across genotypes. Hierarchical clustering was performed using Euclidean distance and complete linkage to identify compositional similarities among hop materials.

The heatmap (Figure 3) illustrates the relative distribution of phenolic acids, flavonoids, and prenylated flavonoids across hop genotypes, revealing clear compositional clustering patterns. Two main groups were observed, reflecting contrasting phenolic allocation strategies. The first cluster was characterized by higher relative abundances of phenolic acids and flavan-3-ols, including genotypes such as La Unión, Ranco, Cluster, and Styrian Golding, which showed consistently elevated levels of compounds such as gallic, protocatechuic, and caffeic acids. These genotypes were associated with lower relative contributions of prenylated flavonoids. In contrast, a second cluster grouped genotypes with higher relative accumulation of prenylated flavonoids, including Vanguard, Nugget, Tahoma, and Glacier, which showed strong enrichment in xanthohumol, desmethylxanthohumol, and prenylnaringenins. These genotypes exhibited comparatively lower levels of phenolic acids. Intermediate profiles were observed in genotypes such as Cascade, Magnum, and Northern Brewer, which displayed more balanced distributions between phenolic acids and prenylated flavonoids. Overall, the heatmap highlights a clear separation between phenolic acid-dominated and prenylated flavonoid-dominated profiles, supporting the patterns observed in the PCA and chemical index analyses. These results reinforce the existence of genotype-dependent metabolic allocation strategies shaping the phenolic composition of hop germplasm cultivated under uniform conditions.

### 3.6. ORAC Analysis

**Figure 4:**
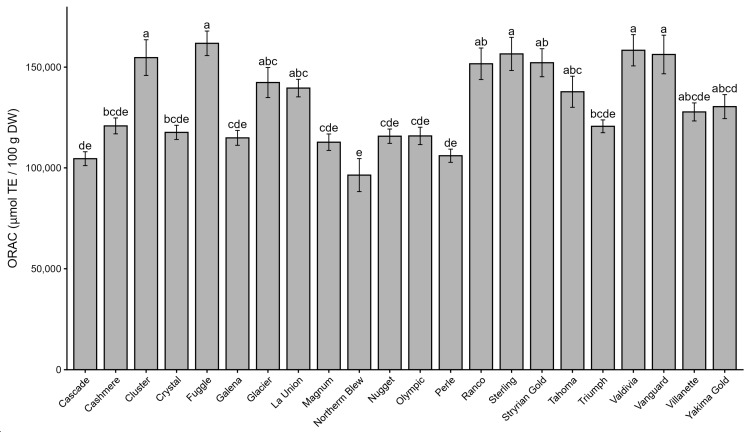
Antioxidant capacity (ORAC) in hop varieties and local ecotypes. Values are expressed as mean ± standard error (SE) (*n* = 3) and reported as µmol Trolox equivalents per 100 g dry weight (µmol TE 100 g^−1^ DW). Different letters above the bars indicate significant differences among hop genotypes according to one-way ANOVA followed by Tukey’s multiple comparison test (*p* < 0.05). Finally, Pearson correlation analysis revealed that ORAC values were strongly associated with condensed tannins (r = 0.774, *p* < 0.001) and total phenolic content (r = 0.739, *p* < 0.001). In contrast, total flavonoids were not significantly correlated with antioxidant capacity (r = 0.205, *p* > 0.05). Notably, total prenylated flavonoids showed a significant negative correlation with ORAC (r = −0.477, *p* < 0.05), which was also observed for individual compounds such as xanthohumol (r = −0.464, *p* < 0.05) and desmethylxanthohumol (r = −0.442, *p* < 0.05). These results suggest that antioxidant capacity in hop genotypes is primarily driven by condensed tannins rather than prenylated flavonoids (Table 5).

Antioxidant capacity, as determined by the ORAC assay, showed significant variation among hop genotypes (F = 10.53, *p* < 0.0001; Table 1). ORAC values ranged from 96,405.31 ± 24,579.86 to 161,815.29 ± 14,942.91 µmol TE 100 g^−1^ DW, indicating substantial variability across the evaluated materials. The highest antioxidant capacity was observed in Fuggle (161,815.29 ± 14,942.91 µmol TE 100 g^−1^ DW), followed by Valdivia (ecotype), Sterling, and Vanguard, all exceeding 156,000 µmol TE 100 g^−1^ DW. In contrast, the lowest value was recorded in Northern Brewer (96,405.31 ± 24,579.86 µmol TE 100 g^−1^ DW). Most genotypes exhibited intermediate values, spanning both commercial cultivars and local ecotypes, reflecting a broad range of antioxidant performance. Although a general association between antioxidant capacity and total phenolic content was observed, this relationship was not strictly proportional, indicating that antioxidant capacity depends not only on total phenolic concentration but also on the relative distribution of phenolic subclasses. Comparisons between commercial cultivars and local ecotypes revealed partially overlapping ranges; however, certain ecotypes, particularly Valdivia and Ranco, exhibited comparatively high antioxidant capacity, highlighting genotype-dependent differences. These patterns are consistent with the multivariate analysis (Figure 4), where antioxidant capacity is associated with specific compositional features rather than bulk phenolic content alone. This interpretation is further supported by Pearson correlation analysis, which revealed that ORAC values were strongly associated with condensed tannins and total phenolic content, whereas total flavonoids showed no significant relationship. Notably, prenylated flavonoids exhibited a negative association with ORAC, indicating that antioxidant capacity is primarily driven by specific phenolic subclasses rather than total flavonoid concentration.

**Table 5:** Pearson correlation coefficients (r) between ORAC and phenolic compounds.

Variable	r	*p*-Value
Condensed tannins	0.774 **	<0.001
Total phenolics	0.739 **	<0.001
Total prenylated flavonoids	−0.477 *	0.025
Xanthohumol	−0.464 *	0.030
Desmethylxanthohumol	−0.442 *	0.039
Prenylnaringenin (6-PN)	0.228 ns	0.308
Total flavonoids	0.205 ns	0.359
Prenylnaringenin (8-PN)	−0.166 ns	0.459
Isoxanthohumol	0.018 ns	0.937

** *p* < 0.001; * *p* < 0.05; ns: not significant.

## 4. Discussion

This study provides a genotype-focused assessment of antioxidant-related phenolic signatures in commercial hop varieties and Chilean local ecotypes cultivated under uniform agronomic and environmental conditions in southern Chile. Because all accessions were grown under homogeneous edaphoclimatic management, the compositional differences observed in total phenolics, flavonoids, condensed tannins, and HPLC-resolved phenolic classes can be largely associated with genotype-dependent chemical differentiation rather than environmental variability. This interpretation aligns with previous evidence demonstrating that hop phenolic composition is strongly influenced by cultivar identity, affecting both total phenolic abundance and the qualitative distribution of phenolic subclasses [20]. In this context, the variability observed among both international cultivars and local ecotypes supports the presence of distinct antioxidant-related chemical phenotypes within hop germplasm cultivated in Chile. This interpretation is further supported by Betancur et al. [26], who demonstrated that Chilean hop ecotypes exhibit high bioactive potential, including strong antioxidant, antimicrobial, and cytotoxic activities, with marked differences among ecotypes. Spectrophotometric parameters and condensed tannin determination provide useful descriptors of bulk phenolic composition; however, they should be interpreted as compositional descriptors rather than direct measures of antioxidant activity. Recent hop-focused studies emphasize that antioxidant-related properties are driven not only by total phenolic content but also by the relative contribution of specific phenolic subclasses, including phenolic acids, flavonoids, and particularly prenylated flavonoids [29,34,35]. Accordingly, integrating bulk spectrophotometric metrics with compound-resolved HPLC profiling enables a more comprehensive characterization of phenolic signatures. In agreement with this, Pearson correlation analysis revealed that antioxidant capacity (ORAC) was strongly associated with condensed tannins and total phenolic content, whereas total flavonoids showed no significant relationship. This indicates that antioxidant capacity in hop genotypes is primarily driven by specific phenolic subclasses rather than total flavonoid concentration alone. HPLC profiling further highlighted that phenolic acids and non-prenylated flavonoids constitute an important axis of chemical differentiation among hop genotypes. These compounds fall within the compositional range commonly reported for hop cones and are known to vary significantly depending on cultivar identity [36,37]. Their contribution to the overall phenolic matrix is therefore relevant when considering compositional diversity and potential functionality. A defining feature of hop chemistry is the presence of prenylated flavonoids, particularly xanthohumol and its derivatives. The marked variability observed among genotypes in prenylated flavonoid accumulation reflects genotype-specific regulation of phenylpropanoid and prenylation pathways, which shape the final phenolic profile. While xanthohumol has been widely reported as a major bioactive compound in hops [38,39,40], its relevance in the present study lies primarily in its contribution to compositional differentiation among genotypes rather than pharmacokinetic considerations. Interestingly, correlation analysis revealed that total prenylated flavonoids and key compounds such as xanthohumol and desmethylxanthohumol were negatively associated with ORAC values. This suggests that prenylated flavonoids do not substantially contribute to peroxyl radical scavenging capacity under the conditions evaluated and may instead be linked to alternative biological functions, such as antimicrobial activity or ecological interactions. This interpretation is further supported by Betancur et al. [26], who identified xanthohumol as a major bioactive compound in Chilean hop ecotypes, particularly in the Valdivia ecotype, where elevated concentrations were associated with enhanced antioxidant and antimicrobial activity. The chemical indices proposed in this study provide an integrative approach to interpreting multidimensional phenolic data. By expressing prenylation and phenolic acid indices, the analysis shifts from absolute concentration comparisons toward relative compositional balance, offering insight into genotype-specific metabolic allocation strategies. This approach is supported by previous studies indicating that different phenolic subclasses contribute unevenly to antioxidant-related properties, even when total phenolic levels are comparable [41,42,43]. However, these indices should be interpreted as comparative descriptors rather than absolute measures, given that they integrate variables obtained from different analytical approaches. Multivariate analysis further supported these compositional patterns by revealing structured variation among genotypes based on phenolic subclasses. Importantly, PCA should be interpreted as a tool for describing compositional differentiation rather than as a direct indicator of antioxidant activity. The dispersion of genotypes across the PCA space reflects differences in phenolic allocation patterns, which help explain the variability observed in antioxidant capacity. This is consistent with the correlation results, which indicate that antioxidant capacity is more strongly associated with specific phenolic subclasses than with total phenolic content alone. Notably, the wide distribution of Chilean ecotypes within the multivariate space highlights their substantial chemical diversity. Among these, the Valdivia ecotype consistently exhibited elevated levels of multiple phenolic compounds, including both bulk phenolics and specific HPLC-resolved metabolites. This profile is consistent with its high antioxidant capacity, which can be mechanistically explained by its elevated condensed tannin content. This compositional profile suggests that certain Chilean ecotypes represent valuable and underexplored sources of phenolic-rich germplasm. This finding is in strong agreement with Betancur et al. [26], who consistently reported the Valdivia ecotype as the most bioactive among Chilean hop materials, exhibiting the highest antioxidant capacity across multiple assays and superior antimicrobial performance. From an applied perspective, the direct comparison between commercial cultivars and local ecotypes under identical growing conditions provides a robust framework for identifying genotypes with differentiated chemical profiles. Recent literature highlights increasing interest in hops as sources of bioactive compounds beyond brewing, with prenylated flavonoids playing a central role in functional ingredient development [44,45,46,47,48]. Within this context, Chilean ecotypes may represent strategic resources for both extract production and breeding programs targeting antioxidant-related traits. In line with this, Betancur et al. [26] demonstrated that extracts from Chilean ecotypes, particularly Valdivia, exhibit multifunctional bioactivity, including antioxidant, antimicrobial, and cytotoxic effects, reinforcing their potential for application in functional foods and nutraceutical development. Overall, the integration of spectrophotometric descriptors, targeted HPLC profiling, antioxidant capacity (ORAC), and compositional indices provides a comprehensive framework for identifying hop genotypes with high potential for antioxidant-oriented applications. Importantly, the present results demonstrate that antioxidant capacity is primarily associated with condensed tannins and total phenolic content, whereas prenylated flavonoids contribute more to compositional differentiation than to direct antioxidant activity.

## 5. Conclusions

This study demonstrates pronounced genotype-dependent variation in antioxidant-related phenolic composition among commercial hop varieties and Chilean local ecotypes cultivated under uniform conditions in southern Chile. Total phenolics, total flavonoids, and condensed tannins exhibited wide quantitative differences, with values ranging from 4051 to 8124 mg GAE/100 g dry weight, 655 to 3011 mg CE/100 g, and 11.0 to 60.1 mg CE/g, respectively. Antioxidant capacity, evaluated by ORAC, ranged from 96,405 to 161,815 µmol TE/100 g dry weight. HPLC profiling revealed marked genotype-driven variation in phenolic acids and prenylated flavonoids. The integration of compositional chemical indices (prenylation and phenolic acid indices) with multivariate analysis confirmed the presence of distinct phenolic signatures across hop materials, reflecting differential allocation among phenolic subclasses rather than total phenolic content alone. Importantly, correlation analysis revealed that antioxidant capacity was strongly associated with condensed tannins and total phenolic content, whereas total flavonoids showed no significant relationship, and prenylated flavonoids were negatively associated with ORAC values. Notably, certain Chilean ecotypes, particularly Valdivia, consistently ranked among the top-performing genotypes for several phenolic traits and showed high antioxidant capacity, highlighting the relevance of local germplasm as a valuable source of bioactive compounds. This superior performance can be attributed to their elevated levels of condensed tannins and overall phenolic content rather than to prenylated flavonoids. These findings support the selection of hop genotypes not only for traditional brewing purposes but also for the development of phenolic-rich ingredients with potential functional applications. More specifically, the results highlight the importance of targeting phenolic subclasses, particularly condensed tannins, when selecting genotypes for antioxidant-related applications. Future research should address biological activity and genotype × environment stability across seasons to validate the robustness and applicability of these compositional traits.

## Figures and Tables

**Table 1:** Summary of one-way ANOVA results for antioxidant capacity, phenolic composition, and derived chemical indices in hop varieties and local ecotypes.

Variable/Compounds	DF (Between)	DF (Within)	F Value	*p*-Value
ORAC	21	44	10.53	<0.0001
TPC	21	44	820	<0.0001
TFC	21	44	310	<0.0001
Condensed tannins	21	44	62.96	<0.0001
Prenylated index	21	44	11,700	<0.0001
Phenolic acid index	21	44	8600	<0.0001

Degrees of freedom (DF), F-values, and corresponding *p*-values are presented for each variable. Statistical analysis was performed to evaluate differences among hop genotypes. All variables showed significant differences (*p* < 0.0001).

**Table 2:** Total phenolics, total flavonoids and condensed tannins in hop varieties and local ecotypes.

Hop Material	Total Phenolics (mg GAE/100 g DW)	Total Flavonoids (mg QE/100 g DW)	Condensed Tannins (mg CE/g DW)
Cascade	4865.36 ± 31.28 i	1902.40 ± 41.45 f	23.84 ± 1.34 e
Cashmere	5039.26 ± 55.46 h	2670.05 ± 24.50 b	26.03 ± 0.37 d
Cluster	7206.01 ± 0.00 c	2016.90 ± 61.87 e	56.29 ± 0.53 a
Crystal	4698.06 ± 42.96 i	1949.70 ± 22.94 e	37.22 ± 1.33 c
Fuggle	6357.30 ± 26.50 e	1789.08 ± 32.42 f	41.91 ± 1.15 c
Glacier	7030.98 ± 110.62 c	3011.28 ± 21.47 a	37.96 ± 0.68 c
Magnum	4923.48 ± 96.55 i	1464.60 ± 25.89 g	20.54 ± 0.78 e
Northern Brewer	6099.85 ± 13.61 f	969.54 ± 27.36 h	21.65 ± 0.66 e
Nugget	4050.98 ± 32.13 j	655.27 ± 15.55 i	12.95 ± 0.48 f
Olympic	6085.62 ± 14.51 f	2336.89 ± 24.23 c	32.47 ± 1.40 c
Perle	4429.12 ± 37.96 j	663.59 ± 11.57 i	13.02 ± 0.34 f
Sterling	6252.78 ± 26.65 e	2810.74 ± 34.16 b	33.64 ± 1.01 c
Styrian Golding	7539.76 ± 25.54 b	2388.04 ± 12.64 c	21.17 ± 0.25 e
Tahoma	5499.59 ± 19.07 g	2947.80 ± 12.08 a	43.83 ± 2.55 b
Triumph	4252.34 ± 29.58 j	858.65 ± 11.74 h	14.05 ± 0.20 f
Vanguard	5583.42 ± 18.96 g	2818.43 ± 34.28 b	31.70 ± 0.77 c
Villanette	6486.13 ± 36.53 e	1931.32 ± 64.79 e	23.72 ± 1.44 e
Yakima Gold	4941.62 ± 58.34 i	949.57 ± 11.55 h	10.98 ± 0.47 f
Galena (ecotype)	5465.37 ± 25.93 g	2008.77 ± 57.72 e	46.49 ± 1.08 b
La Unión (ecotype)	6201.72 ± 130.66 e	2171.91 ± 47.24 d	33.57 ± 0.74 c
Ranco (ecotype)	6839.48 ± 98.24 d	1835.95 ± 42.83 f	41.40 ± 0.83 c
Valdivia (ecotype)	8123.83 ± 65.48 a	2349.78 ± 24.61 c	60.05 ± 0.81 a

Values are expressed as mean ± standard error (SE) (*n* = 3). Statistical comparisons were performed independently for each variable. Different letters within each column indicate significant differences among hop materials according to one-way ANOVA followed by Tukey’s multiple comparison test (*p* < 0.05).

**Table 3:** Phenolic acids and individual flavonoids identified and quantified by HPLC–DAD in hop varieties and local ecotypes.

Hop Material	Gallic Acid	Protocatechuic Acid	Catechin	Caffeic Acid	Vanillic Acid	Syringic Acid	p-Coumaric Acid	Ellagic Acid
Cascade	0.34 ± 0.01 c	0.41 ± 0.02 d	0.52 ± 0.02 d	0.29 ± 0.01 c	0.18 ± 0.01 d	0.21 ± 0.01 c	0.09 ± 0.00 d	0.14 ± 0.01 c
Cashmere	0.29 ± 0.01 d	0.37 ± 0.01 e	0.44 ± 0.01 e	0.25 ± 0.01 d	0.16 ± 0.01 e	0.19 ± 0.01 d	0.08 ± 0.00 e	0.12 ± 0.01 d
Cluster	0.51 ± 0.02 a	0.62 ± 0.02 a	0.81 ± 0.03 a	0.46 ± 0.02 a	0.33 ± 0.01 a	0.35 ± 0.01 a	0.21 ± 0.01 a	0.29 ± 0.01 a
Crystal	0.33 ± 0.01 c	0.44 ± 0.01 d	0.56 ± 0.02 c	0.31 ± 0.01 c	0.20 ± 0.01 c	0.22 ± 0.01 c	0.10 ± 0.00 d	0.15 ± 0.01 c
Fuggle	0.47 ± 0.02 b	0.55 ± 0.02 b	0.72 ± 0.02 b	0.41 ± 0.02 b	0.29 ± 0.01 b	0.31 ± 0.01 b	0.18 ± 0.01 b	0.24 ± 0.01 b
Glacier	0.21 ± 0.01 e	0.29 ± 0.01 f	0.38 ± 0.01 f	0.19 ± 0.01 e	0.12 ± 0.01 f	0.15 ± 0.01 e	0.06 ± 0.00 f	0.09 ± 0.00 e
Magnum	0.31 ± 0.01 d	0.39 ± 0.01 e	0.49 ± 0.02 d	0.27 ± 0.01 d	0.17 ± 0.01 d	0.20 ± 0.01 d	0.09 ± 0.00 d	0.13 ± 0.01 d
Northern Brewer	0.26 ± 0.01 e	0.34 ± 0.01 f	0.41 ± 0.01 f	0.23 ± 0.01 e	0.14 ± 0.01 f	0.17 ± 0.01 e	0.07 ± 0.00 e	0.11 ± 0.01 e
Nugget	0.19 ± 0.01 f	0.27 ± 0.01 g	0.33 ± 0.01 g	0.17 ± 0.01 f	0.10 ± 0.00 g	0.13 ± 0.01 f	0.05 ± 0.00 f	0.08 ± 0.00 f
Olympic	0.42 ± 0.02 b	0.51 ± 0.02 b	0.69 ± 0.02 b	0.39 ± 0.02 b	0.27 ± 0.01 b	0.29 ± 0.01 b	0.16 ± 0.01 b	0.22 ± 0.01 b
Perle	0.18 ± 0.01 f	0.25 ± 0.01 g	0.31 ± 0.01 g	0.16 ± 0.01 f	0.09 ± 0.00 g	0.12 ± 0.01 f	0.05 ± 0.00 f	0.07 ± 0.00 f
Sterling	0.37 ± 0.01 c	0.46 ± 0.02 c	0.61 ± 0.02 c	0.34 ± 0.01 c	0.23 ± 0.01 c	0.25 ± 0.01 c	0.13 ± 0.01 c	0.18 ± 0.01 c
Styrian Golding	0.50 ± 0.02 a	0.60 ± 0.02 a	0.79 ± 0.03 a	0.45 ± 0.02 a	0.32 ± 0.01 a	0.34 ± 0.01 a	0.21 ± 0.01 a	0.28 ± 0.01 a
Tahoma	0.23 ± 0.01 e	0.31 ± 0.01 f	0.40 ± 0.01 f	0.21 ± 0.01 e	0.13 ± 0.01 f	0.16 ± 0.01 e	0.06 ± 0.00 f	0.10 ± 0.01 e
Triumph	0.20 ± 0.01 f	0.28 ± 0.01 g	0.35 ± 0.01 g	0.18 ± 0.01 f	0.11 ± 0.00 g	0.14 ± 0.01 f	0.05 ± 0.00 f	0.09 ± 0.00 f
Vanguard	0.28 ± 0.01 d	0.36 ± 0.01 e	0.46 ± 0.01 e	0.26 ± 0.01 d	0.16 ± 0.01 e	0.19 ± 0.01 d	0.08 ± 0.00 e	0.12 ± 0.01 d
Villanette	0.35 ± 0.01 c	0.43 ± 0.02 d	0.57 ± 0.02 c	0.32 ± 0.01 c	0.21 ± 0.01 c	0.23 ± 0.01 c	0.11 ± 0.01 d	0.16 ± 0.01 c
Yakima Gold	0.27 ± 0.01 e	0.35 ± 0.01 f	0.42 ± 0.01 f	0.24 ± 0.01 e	0.15 ± 0.01 f	0.18 ± 0.01 e	0.07 ± 0.00 e	0.11 ± 0.01 e
Galena (ecotype)	0.39 ± 0.01 c	0.48 ± 0.02 c	0.63 ± 0.02 c	0.36 ± 0.01 c	0.24 ± 0.01 c	0.26 ± 0.01 c	0.14 ± 0.01 c	0.19 ± 0.01 c
La Unión (ecotype)	0.49 ± 0.02 a	0.58 ± 0.02 a	0.78 ± 0.03 a	0.44 ± 0.02 a	0.31 ± 0.01 a	0.33 ± 0.01 a	0.20 ± 0.01 a	0.27 ± 0.01 a
Ranco (ecotype)	0.46 ± 0.02 b	0.54 ± 0.02 b	0.74 ± 0.03 b	0.42 ± 0.02 b	0.30 ± 0.01 b	0.32 ± 0.01 b	0.19 ± 0.01 b	0.25 ± 0.01 b
Valdivia (ecotype)	0.52 ± 0.02 a	0.63 ± 0.02 a	0.83 ± 0.03 a	0.47 ± 0.02 a	0.34 ± 0.01 a	0.36 ± 0.01 a	0.22 ± 0.01 a	0.30 ± 0.01 a

Values are expressed as mean ± standard error (SE) (*n* = 3). Statistical comparisons were performed independently for each compound. Different letters within each column indicate significant differences among hop materials according to one-way ANOVA followed by Tukey’s multiple comparison test (*p* < 0.05). Results are expressed on a dry weight basis (mg g^−1^ DW).

**Table 4:** Prenylated flavonoids and naringenin quantified by HPLC–DAD in hop varieties and local ecotypes.

Hop Material	Xanthohumol	Desmethylxanthohumol	Isoxanthohumol	8-Prenylnaringenin	6-Prenylnaringenin	Naringenin
Cascade	3.06 ± 0.04 d	1.43 ± 0.02 b	0.08 ± 0.00 d	0.09 ± 0.00 e	ND	0.01 ± 0.00 d
Cashmere	3.22 ± 0.04 c	0.74 ± 0.01 d	0.01 ± 0.00 e	0.15 ± 0.00 d	0.01 ± 0.00 b	0.01 ± 0.00 d
Cluster	0.63 ± 0.01 g	0.15 ± 0.00 f	0.00 ± 0.00 f	0.01 ± 0.00 f	ND	ND
Crystal	3.44 ± 0.05 c	1.35 ± 0.02 b	0.06 ± 0.00 d	ND	0.01 ± 0.00 b	0.07 ± 0.00 b
Fuggle	2.55 ± 0.03 e	0.46 ± 0.01 e	0.06 ± 0.00 d	0.09 ± 0.00 e	ND	ND
Glacier	2.38 ± 0.03 e	0.70 ± 0.01 d	0.08 ± 0.00 d	0.61 ± 0.01 a	0.03 ± 0.00 a	0.01 ± 0.00 d
Magnum	4.83 ± 0.06 b	1.37 ± 0.02 b	0.07 ± 0.00 d	0.18 ± 0.00 c	ND	0.02 ± 0.00 c
Northern Brewer	2.51 ± 0.03 e	0.98 ± 0.01 c	0.07 ± 0.00 d	0.06 ± 0.00 e	0.01 ± 0.00 b	0.04 ± 0.00 b
Nugget	1.52 ± 0.02 f	0.50 ± 0.01 e	0.55 ± 0.01 a	0.63 ± 0.01 a	0.07 ± 0.00 a	0.02 ± 0.00 c
Olympic	2.73 ± 0.04 d	1.13 ± 0.02 c	0.15 ± 0.00 c	0.23 ± 0.00 c	ND	ND
Perle	1.84 ± 0.02 f	0.41 ± 0.01 e	0.01 ± 0.00 e	0.31 ± 0.00 b	ND	ND
Sterling	3.13 ± 0.04 c	1.01 ± 0.01 c	0.08 ± 0.00 d	0.09 ± 0.00 e	ND	0.01 ± 0.00 d
Styrian Golding	2.99 ± 0.04 d	1.06 ± 0.01 c	0.06 ± 0.00 d	0.03 ± 0.00 f	0.01 ± 0.00 b	0.01 ± 0.00 d
Tahoma	2.28 ± 0.03 e	1.23 ± 0.02 b	0.36 ± 0.01 b	0.24 ± 0.00 c	0.07 ± 0.00 a	0.03 ± 0.00 c
Triumph	2.21 ± 0.03 e	0.92 ± 0.01 c	0.11 ± 0.00 c	0.20 ± 0.00 c	ND	ND
Vanguard	6.01 ± 0.07 a	2.04 ± 0.02 a	0.51 ± 0.01 a	0.28 ± 0.00 c	0.07 ± 0.00 a	0.04 ± 0.00 b
Villanette	2.16 ± 0.03 e	0.73 ± 0.01 d	0.05 ± 0.00 d	0.05 ± 0.00 e	ND	ND
Yakima Gold	0.51 ± 0.01 g	0.22 ± 0.00 f	0.13 ± 0.00 c	0.33 ± 0.00 b	ND	ND
Galena (ecotype)	0.88 ± 0.01 f	0.15 ± 0.00 f	0.43 ± 0.01 b	0.13 ± 0.00 d	ND	ND
La Unión (ecotype)	0.99 ± 0.01 f	0.32 ± 0.01 f	0.55 ± 0.01 a	0.39 ± 0.01 b	ND	ND
Ranco (ecotype)	0.49 ± 0.01 g	0.23 ± 0.00 f	0.61 ± 0.01 a	0.61 ± 0.01 a	0.01 ± 0.00 b	ND
Valdivia (ecotype)	1.72 ± 0.02 f	0.48 ± 0.01 e	0.40 ± 0.01 b	0.22 ± 0.00 c	ND	ND

Values are expressed as mean ± standard error (SE) (*n* = 3). Statistical comparisons were performed independently for each compound. Different letters within each column indicate significant differences among hop materials according to one-way ANOVA followed by Tukey’s multiple comparison test (*p* < 0.05). Results are expressed on a dry weight basis (mg g^−1^ DW). ND indicates compounds not detected under the analytical conditions used.

## Data Availability

The raw data supporting the conclusions of this article will be made available by the authors on request.
